# Mild pulmonary hemodynamic alterations in patients with systemic sclerosis: relevance of the new 2022 ESC/ERS definition of pulmonary hypertension and impact on mortality

**DOI:** 10.1186/s12931-022-02205-4

**Published:** 2022-10-15

**Authors:** Sébastien Puigrenier, Jonathan Giovannelli, Nicolas Lamblin, Pascal De Groote, Marie Fertin, Jean-François Bervar, Antoine Lamer, Jean-Louis Edmé, Marie-Hélène Balquet, Vincent Sobanski, David Launay, Éric Hachulla, Sébastien Sanges

**Affiliations:** 1 CHU Lille, Service de Médecine Interne Et Immunologie Clinique, Centre de Référence Des Maladies Auto-Immunes Systémiques Rares du Nord Et Nord-Ouest de France (CeRAINO), 59000 Lille, France; 2 Health Care Provider of the European Reference Network on Rare Connective Tissue and Musculoskeletal Diseases Network (ReCONNET), 59000 Lille, France; 3 CH Boulogne-Sur-Mer, Service de Médecine Et Néphrologie, 62200 Boulogne-Sur-Mer, France; 4 GIOVANNELLI Epidemiology and Clinical Research Counselling, Lille, France; 5 CHU Lille, Service de Cardiologie, 59000 Lille, France; 6 Institut Pasteur de Lille, Inserm U1167, 59000 Lille, France; 7 CHU Lille, Service de Pneumologie, 59000 Lille, France; 8 Univ. Lille, CHU Lille, ULR 2694-METRICS: Évaluation Des Technologies de Santé Et Des Pratiques Médicales, 59000 Lille, France; 9 Service Des Explorations Fonctionnelles Respiratoires, CHU Lille, 59000 Lille, France; 10 EA 4483-IMPECS-IMPact de L’Environnement Chimique Sur La Santé Humaine, Univ. Lille, 59000 Lille, France; 11 CH Lens, Service de Médecine Interne, 62300 Lens, France; 12 Univ. Lille, U1286 - INFINITE - Institute for Translational Research in Inflammation, 59000 Lille, France; 13 59000 Lille, France

**Keywords:** Systemic sclerosis, Pulmonary hypertension, Pulmonary arterial hypertension, Pulmonary vascular resistance, Diagnostic criteria, Mortality

## Abstract

**Background and objective:**

The definition of pre-capillary pulmonary hypertension (PH) has been modified, with lowering of the mean pulmonary arterial pressure (mPAP) threshold from 25 to 20 mmHg and addition of a mandatory criterion of pulmonary vascular resistance (PVR) ≥ 2 Wood units (WU). Our objectives were: 1/ to estimate the proportion of patients reclassified as having pre-capillary PH when using the new 2022 ESC/ERS hemodynamic criteria (i.e. mPAP 21-24 mmHg and PVR ≥ 2 WU), and to describe their clinical characteristics and outcome; and 2/ to study the relationship between PVR and survival in patients with mPAP > 20 mmHg.

**Methods:**

We retrospectively analyzed consecutive SSc patients included in our National Reference Center for a first right-heart catheterization between 2003 and 2018. The association between survival and PVR was studied using smoothing splines.

**Results:**

We included 126 SSc patients with mPAP > 20 mmHg. Among them, 16 (13%) had a baseline mPAP value between 21 and 24 mmHg and PVR ≥ 2 mmHg and were reclassified as pre-capillary PH; 10 of which (62%) raised their mPAP ≥ 25 mmHg during follow-up. In patients with mPAP > 20 mmHg, we observed a linear relation between PVR and mortality for values < 6 WU.

**Conclusion:**

A significant proportion of SSc patients is reclassified as having pre-capillary PH with the new 2022 ESC/ERS hemodynamic definition. Lowering the PVR threshold from 3 to 2 WU captures patients at risk of raising their mPAP > 25 mmHg, with a possibly less severe disease.

**Supplementary Information:**

The online version contains supplementary material available at 10.1186/s12931-022-02205-4.

## Introduction

Pulmonary hypertension (PH) is one of the most severe complications of systemic sclerosis (SSc), affecting more than 10% of patients during the course of the disease [1]. PH in SSc may result from several mechanisms: group 1 due to pulmonary microangiopathy (*i.e.* pulmonary arterial hypertension (PAH)), group 1’ due to pulmonary veno-occlusive disease (PVOD), group 2 due to myocardial fibrosis with left heart dysfunction, and/or group 3 due to severe interstitial lung disease (ILD). Its prognosis remains poor despite therapeutic progresses [2, 3].

The diagnosis of PH is based on right-heart catheterization (RHC). A new hemodynamic definition of pre-capillary PH was proposed in the 2022 European Society of Cardiology (ESC)/European Respiratory Society (ERS) guidelines [4]. The mean pulmonary arterial pressure (mPAP) threshold was lowered from 25 to 20 mmHg in combination with pulmonary arterial wedge pressure (PAWP) ≤ 15 mmHg; and a mandatory criterion of pulmonary vascular resistance (PVR) ≥ 2 Wood units (WU) was added.

This modified definition of pre-capillary PH is debated in the field of SSc because of several unresolved issues. First, the impact of these revised criteria on the number of patients reclassified as PH is has not been extensively studied. Indeed, previous works [5–7] focusing on this issue have used the provisional hemodynamic definition from the 6^th^ World Symposium on PH (WSPH) [8] that set the PVR threshold at 3 WU. Only one study used the current PVR cut-off of 2 WU and suggested a proportion of 9.85% PH-reclassified patients [6]. Second, the optimal PVR cut-off associated with increased mortality is controversial [6].

In order to add data and better decipher the impact of the new definition in SSc, we performed a retrospective monocentric study on our cohort of SSc patients with mPAP > 20 mmHg on their first-ever RHC. Our objectives were: 1/ to estimate the proportion of patients reclassified as having pre-capillary PH when using the new hemodynamic definition (*i.e.* mPAP between 21 and 24 mmHg and PVR ≥ 2 WU), and to describe their clinical characteristics and outcome; and 2/ to study the relationship between PVR and survival in patients with mPAP > 20 mmHg.

## Methods

### Study population

Consecutive patients were recruited from our National Reference Center for SSc. They were included in the study if they met the following criteria: 1/ a definite diagnosis of SSc (diffuse cutaneous SSc (dc-SSc) or limited cutaneous SSc (lc-SSc)) according to the 2013 American College of Rheumatology/European League against Rheumatism classification criteria [9] 2/ a first-ever RHC performed between 01/01/2003 and 31/12/2018; 3/ an mPAP value > 20 mmHg; 4/ an age ≥ 18 years old.

### Study measurements

Data were retrospectively collected at the baseline visit (which was defined as the visit of the first RHC) and comprised clinical characteristics (including SSc subtype, New York Heart Association (NYHA) functional class, presence of interstitial lung disease (ILD) and 6-min walking distance (6MWD)), echocardiography parameters (tricuspid regurgitation velocity (TRV), right ventricle (RV)/left ventricle (LV) ratio, right atrium (RA) area, pulmonary artery acceleration time (PAAT), tricuspid annular plane systolic excursion (TAPSE), inferior vena cava (IVC) diameter), E/A ratio, left atrium (LA) area and LV ejection fraction (LVEF)), biological results (including brain natriuretic peptide (BNP) levels), electrocardiogram (ECG), pulmonary function tests (PFT) results, RHC parameters and initial treatment strategy. Data were also collected at last follow-up and included last treatment strategy and dead-or-alive status.

RHC were performed by pulmonary hypertension specialist practitioners using a 6-French Swan-Ganz catheter in non-acute clinical conditions. The catheter was placed under fluoroscopic guidance from a right antebrachial vein or a right femoral vein as needed. The correct position of the catheter was confirmed by fluoroscopy and by the presence of characteristic pressure waveforms. The “zero-ing” transducer to atmospheric pressure was at the level of the mid-axillary line. Right atrial pressure (RAP), mPAP and PAWP were determined electronically by the mean of several beats during quiet breathing. mPAP was determined by the mean of the area under pressure curves. Cardiac Output (CO) was measured by the thermodilution technique (mean of 3–6 measures with exclusion of measurements that exceed 10% of the mean); and Cardiac Index (CI) calculated by the ratio of CO to body surface area. Mixed venous oxygen saturation (SvO_2_) was measured from blood drawn from the pulmonary artery. PVR were then calculated (PVR = [mPAP-PAWP]/CO). In case of mPAP > 20 mmHg and a PAWP more than 12 mmHg (13–15 mmHg), suggesting a possible post-capillary pulmonary hypertension, a fluid challenge (500 mL of saline solution over 5 min) was performed as proposed in guidelines [10].

The initial (*i.e.* within the first 4 months of baseline visit) and follow-up treatment strategies were defined according to the number of PAH medications prescribed (endothelin receptor antagonist, phosphodiesterase type 5 inhibitor, and prostacyclin analogue). Patients whose death was not registered were censored at the time of last clinical contact.

### Statistical analysis

The characteristics of the population were described by using the mean ± standard deviation (SD), or median ± interquartile range (IQR) in case of non-normality, for quantitative variables; numbers (percentage) were used for qualitative variables. PVR were categorized in four groups: < 2, 2–3, 3–6 and > 6 WU. No imputation was performed for missing data. Characteristics of patients between these groups were compared by using a one-way analysis of variance (ANOVA), or a Kruskal–Wallis test in cases of non-normality for quantitative variables; Fisher exact test was used for qualitative variables, except for the comparison of treatments (Chi-squared test with estimated *p*-values by Monte Carlo simulation).

To discuss the impact of the new PH definition on our patients, we described the clinical characteristics and outcome of patients reclassified as having pre-capillary PH (*i.e.* patients with mPAP between 21 and 24 mmHg, PVR ≥ 2 WU and PAWP ≤ 15 mmHg) according to the new hemodynamic definition from the 2022 ESC/ERS guidelines.

Survival was right-censored at 5 years and described by using the Kaplan–Meier method, for the whole population and by PVR groups. The relationship between survival and PVR and was assessed by a smoothing spline using a penalized spline basis for predictor, without and with adjustments for age, gender, SSc subtype, date of SSc diagnosis, NYHA class, ILD and follow-up treatment strategy. We also studied the association between survival and PVR groups using Cox proportional hazards models without and with adjustments.

All statistical analyses were performed by using R software, version 3.6.2 (*R Foundation for Statistical Computing*), using “Hmisc” and “survival” packages. The threshold for statistical significance was set to *p* < 0.05.

## Results

### Characteristics of the whole population according to PVR values

**Fig. 1:**
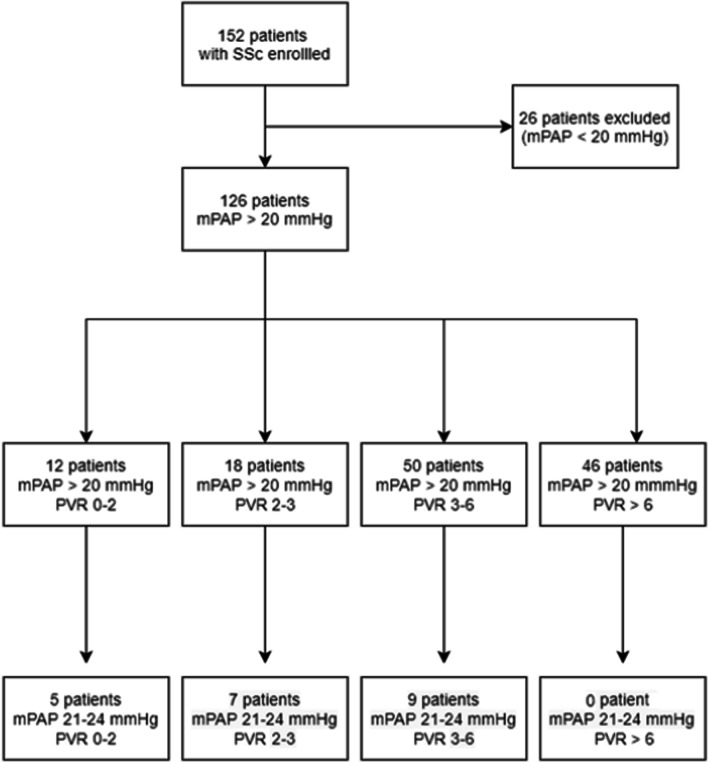
Flow chart of the study

Overall, 126 SSc patients had an mPAP > 20 mmHg on their first-ever RHC and were included in the study (Fig. 1). Patients were classified in 4 groups according to their PVR values: 12, 18, 50 and 46 patients had PVR < 2 WU, between 2–3 WU, between 3 and 6 WU, and > 6 WU, respectively (Table 1).

**Table 1:** Characteristics of the 126 SSc patients with mPAP > 20 mmHg

	N	Whole population (n = 126)	PVR [0—2 WU] (n = 12)	PVR [2—3 WU] (n = 18)	PVR [3—6 WU] (n = 50)	PVR [6—20 WU] (n = 46)	*p*
**SSc characteristics**							
Age (yo) Gender (female) SSc subtype (lcSSc) ACA ATA Time since SSc diagnosis (y) Time since RP onset (y) Time since FEFRPS onset (y) ILD NYHA I—II III-IV 6MWD (m) BNP (ng/L)	126 126 116 107 111 120 98 98 115 101 101 87 75	66.5 ± 16.6 104 (82.5) 95 (81.9) 60 (56.1) 24 (21.6) 7 ± 12 14 ± 15.8 9.5 ± 12.8 54 (47) 41 (40.6) 60 (59.4) 321 ± 173.5 37 ± 36.5	61.2 ± 8 9 (75) 8 (66.7) 3 (27.3) 5 (41.7) 9 ± 11.3 9 ± 10 9 ± 9 8 (66.7) 5 (55.6) 4 (44.4) 310.5 ± 81.8 40 ± 12.5	63.6 ± 18.5 18 (88.9) 16 (100) 12 (70.6) 4 (23.5) 6 ± 9.5 17 ± 12.8 10.5 ± 7.8 5 (31.3) 11 (91.7) 1 (8.3) 388 ± 46.5 53.5 ± 30.8	68.8 ± 13.8 38 (76) 31 (70.5) 21 (55.3) 10 (23.8) 7 ± 13 16 ± 18.8 9 ± 14 23 (50) 19 (47.5) 21 (52.5) 348 ± 116.5 27 ± 27.8	67.1 ± 17.1 41 (89.1) 40 (90.9) 24 (58.5) 5 (12.5) 7 ± 12.5 10 ± 17.8 10 ± 12.5 18 (43.9) 6 (15) 34 (85) 240 ± 139 40 ± 35	0.33 0.27 0.005 0.16 0.17 0.67 0.26 1 0.3 < 0.001 < 0.001 < 0.001 0.23
**RHC parameters**							
mPAP (mmHg) PVR (WU) TPR (WU) RAP (mmHg) sPAP (mmHg) dPAP (mmHg) PAWP (mmHg) CO (L/min) CI (L/min/m^2^) SvO_2_ (%)	126 126 125 121 124 124 126 125 126 82	34.5 ± 16.8 4.9 ± 4.7 7.3 ± 4.9 7 ± 4 54 ± 29.3 19 ± 12.3 10 ± 6 4.7 ± 1.6 2.8 ± 1 68 ± 13.4	26 ± 8.3 1.6 ± 0.2 4.4 ± 3 8 ± 3.5 39 ± 11.5 13 ± 7.3 15 ± 9.8 5.5 ± 1.5 3.1 ± 1.1 70 ± 11.8	26 ± 5.5 2.4 ± 0.4 4.2 ± 1.7 6 ± 4 41 ± 7.5 13 ± 5.8 12 ± 8 6.1 ± 2.3 3.3 ± 0.7 74 ± 3	32.5 ± 8.8 4.4 ± 1.6 6.5 ± 1.7 6 ± 4 50 ± 17 17 ± 7 10 ± 5.8 4.8 ± 1.4 2.8 ± 0.8 68.5 ± 10.8	47.5 ± 11 9.2 ± 3.4 11.9 ± 3.3 8 ± 7 75 ± 21 28 ± 8 8 ± 4 4.2 ± 1.2 2.4 ± 0.7 59.6 ± 11.5	< 0.001 < 0.001 < 0.001 0.004 < 0.001 < 0.001 < 0.001 < 0.001 < 0.001 < 0.001
**Hemodynamic classification**							
Pre-capillary PH Isolated post-capillary PH Combined PH	86 12 10	86 (68.3) 12 (9.5) 10 (7.9)	0 6 (50) 0	12 (66.6) 6 (33.3) 0	44 (88) 0 6 (12)	42 (91.3) 0 4 (8.7)	< 0.001 < 0.001 < 0.001
Pulmonary function tests*							
FVC (% predicted) FEV1 (% predicted) TLC (% predicted) DLCO (% predicted) KCO (% predicted)	95 95 92 91 80	88 ± 23.5 80.9 ± 22.8 84.1 ± 18 41.8 ± 15.8 41.5 ± 12.8	86.9 ± 27 79 ± 23.3 85.4 ± 25 59.1 ± 21.1 55 ± 13.8	91.6 ± 19.8 85.6 ± 17.4 84.1 ± 15.9 53.9 ± 14.9 54 ± 10.5	90.2 ± 25.8 85.3 ± 27.2 83.1 ± 18.9 38.5 ± 14.3 38.9 ± 11.1	84.2 ± 21.4 74.9 ± 18.1 84.9 ± 16.3 38.7 ± 13.2 36.6 ± 10.5	0.66 0.21 0.97 < 0.001 < 0.001
**Echocardiography**							
TRV (m/s) RV/LV ratio PAAT (m/s) RA area (cm^2^) Expiration IVC diam. (mm) TAPSE (mm) LVEF (%) LA area (cm^2^) E/A ratio	84 69 87 94 80 93 101 87 79	3.4 ± 0.8 1 ± 0.4 75 ± 28 18 ± 9.1 15.2 ± 5.8 20.8 ± 5.9 65 ± 5 19.5 ± 8.1 0.8 ± 0.4	3 ± 0.6 0.8 ± 0.2 110 ± 32 16.5 ± 5.6 17.3 ± 5.4 21 ± 7.2 50 ± 15 21.6 ± 8.9 0.9 ± 0.5	3 ± 0.5 0.8 ± 0.2 86.5 ± 38.3 18 ± 4.5 13.5 ± 3 24 ± 6.6 65 ± 5 20.8 ± 5.5 1 ± 0.4	3.4 ± 0.6 0.9 ± 0.2 76 ± 24.5 16.7 ± 8.8 14.9 ± 4.4 21.5 ± 4.7 65 ± 10 19.6 ± 8.5 0.8 ± 0.3	4.1 ± 0.4 1.3 ± 0.2 64 ± 25.5 22.6 ± 9 18.8 ± 4.8 17.7 ± 5.4 65 ± 5 17.1 ± 5 0.7 ± 0.3	< 0.001 < 0.001 0.002 0.014 < 0.001 < 0.001 0.07 0.04 0.004
Therapeutic strategy *							
*Initial* No treatment Monotherapy Dual therapy Triple therapy *Last follow-up* No treatment Monotherapy Dual therapy Triple therapy	126 126	49 (38.9) 51 (40.5) 22 (17.5) 4 (3.2) 36 (28.6) 34 (27) 34 (27) 22 (17.5)	10 (83.3) 2 (16.7) 0 0 8 (66.7) 3 (25) 0 1 (8.3)	15 (83.3) 3 (16.7) 0 0 9 (50) 8 (44.4) 1 (5.6) 0	22 (44) 23 (46) 5 (10) 0 17 (34) 15 (30) 13 (26) 5 (10)	2 (4.4) 23 (50) 17 (37) 4 (8.7) 2 (4.4) 8 (17.4) 20 (43.5) 16 (34.8)	0.001 0.001

6MWD: 6-min walking distance, ACA: anti-centromere antibodies, ATA: anti-topoisomerase antibodies, BNP: brain natriuretic peptide, CI: cardiac index, CO: cardiac output, diam: diameter, DLCO: diffusing capacity of the lung for carbon monoxide, dPAP: diastolic pulmonary arterial pressure, FEFRPS: first except for RP symptom, FEV1: forced expired volume at 1st minute, FVC: forced vital capacity, ILD: interstitial lung disease, IVC: inferior vena cava, KCO: carbon monoxide transfer coefficient, LA: left atrial, lc-SSc: limited cutaneous systemic sclerosis, LVEF: left ventricular ejection fraction, mPAP: mean pulmonary arterial pressure, NYHA: New York Heart Association, PAAT: pulmonary artery acceleration time, PAWP: pulmonary arterial wedge pressure, PVR: pulmonary vascular resistance, RA: right atrium, RAP: right atrial pressure, RHC: right heart catheterization, RP: Raynaud phenomenon, RV/LV: right ventricle / left ventricle, sPAP: systolic pulmonary arterial pressure, SSc: systemic sclerosis, SvO_2_: venous saturation in oxygen, TAPSE: tricuspid annular plane systolic excursion, TLC: total lung capacity, TPR: total pulmonary resistance, TRV: tricuspid regurgitation velocity, y: year, yo: years old

Data were expressed as median ± IQR, except for parameters notified with an asterisk * (mean ± SD)

We did not observe statistical differences in age, gender, antibody profile and disease duration between groups. In terms of PH characteristics, we did not observe significant differences in ILD occurrence and FVC values, indicating similar proportions of group 3 PH between classes; however, PAWP and LVEF differed significantly according to PVR values, consistent with a higher frequency of post-capillary PH (either isolated or combined with pre-capillary PH) in patients with lower PVR (6, 6, 6 and 4 patients with PAWP > 15 mmHg between in the PVR < 2 WU, PVR [2, 3] WU, PVR [3–6] and PVR > 6 WU groups, respectively). Aside from BNP levels, usual markers of PH severity (NYHA class, 6MWD, DLCO, KCO, TRV, RV/LV ratio, PAAT, RA area) were significantly different among groups, with milder anomalies observed with lower PVR. Therapeutic strategies varied with PVR classes, with patients having higher values being more likely to be treated. Only 3 patients were treated by PAH-specific drugs before PAH diagnosis (bosentan prescribed because of digital ulcers in all cases), all of them from the PVR [3–6] group. Triple therapy was started a median of 2 years [1–3] after PH diagnosis.

### Proportion of pre-capillary PH-reclassified SSc patients and population characteristics

Among the 126 included patients, we identified 16 patients (13%) that had a baseline mPAP value between 21 and 24 mmHg, PVR ≥ 2 WU and PAWP ≤ 15 mmHg, and who were thus reclassified as having pre-capillary PH according to the 2022 ESC/ERS hemodynamic definition (Table 2).

**Table 2:** Detailed characteristics of patients with mPAP between 21 and 24 mmHg and PVR ≥ 2 mmHg

Pt #	Patient and SSc characteristics					Clinical characteristics					Lab tests			Chest CT-scan			Pulmonary function tests						
	Sex	Age (yo)	Time since SSc diag/RP/FEFRPS (y)	SSc type	Antibody profile	mRSS	Tel	RHF	NYHA class	6MWD (m) (%pred)	Nt-pro-BNP (ng/L)	BNP (ng/L)	Urate (mg/L)	ILD	CTE	PVOD	FVC (% pred)	FEV1 (% pred)	FEV/FVC (%)	TLC (% pred)	DLCO (% pred)	KCO (% pred)	CVF/ DLCO
1 2 3 4 5 6 7 8 9 10 11 12 13 14 15 16	F F F F F F M F F F F F F F M F	81 71 72 44 82 55 60 65 80 74 52 77 45 65 78 49	0/10/0 17/17/17 15/-/- 1/-/1 1/3/2 7/25/17 1/1/1 2/4/4 1/16/1 22/23/23 11/17/16 22/30/22 30/30/27 6/20/20 7/-/- 2/-/-	— lc lc lc dc lc dc lc lc lc lc lc lc lc lc lc	ACA AFA — ACA No spec ACA ATA ACA ACA ACA ACA ATA ACA ACA ACA ACA	— 2 — 2 23 10 26 — 2 — 6 — 4 2 — —	— Yes — Yes No Yes No — Yes — Yes — Yes No Yes Yes	Yes No No No No No Yes Yes No Yes No No No No No No	— II III III II III II III III — II III II II — II	— 273 (49) 210 (38) 372 (63) 330 (73) 426 (66) 307 (62) 348 (59) 325 (69) — 435 (70) 293 (77) 468 (64) 387 (70) — 390 (56)	— 73 — 67 — 135 — — 689 — — 208 — 67 — —	286 58 130 < 20 114 50 36 — 88 — 57 — 50 59 44 68	79 89 87 46 47 44 46 — 74 — 59 — — 54 59 36	No No No No yes (lim) No yes (ext) yes (ext) yes (lim) — No Yes No No No No	No No — No — No — — No No No — No — No No	— Yes — Yes — No No — No — Yes — Yes — No No	— 98 91 81 113 93 93 80 96 94 115 87 — 120 — —	40 100 92 65 116 65 94 84 86 82 91 91 — 105 — —	57 83 109 86 110 76 106 114 100 96 85 112 — 94 — —	— 92 79 86 81 88 78 62 88 — — — — — — —	— 37 22 28 47 44 28 30 50 66 53 71 — 50 — —	— 43 25 27 63 49 31 40 45 59 44 70 — 43 — —	— 2.6 4.1 2.9 2.4 2.1 3.3 2.7 1.9 1.4 2.2 1.2 — 2.4 — —

2Pt #	ECG	Right-heart catheterization												Echocardiography*												
	RAxD	mPAP (mmHg)	PVR (WU)	**CO** (L/min)	**CI** (L/min/ **m**^**2**^**)**	PAWP (mmHg)	**RAP** **(mmHg)**	**SvO**_**2**_ **(%)**	**HVPG**	Exercise mPAP (mmHg)	Exercise PAWP (mmHg)	Fluid ch mPAP **(mmHg)**	Fluid ch PAWP (mmHg)	TRV (m/s)	RA area **(cm**^**2**^**)**	RV/LV ratio	**PAAT** **(ms)**	IVC diam (mm)	**TAPSE** **(mm)**	RV S’ wave (cm/s)	LVEF (%)	LA vol. index (mL/m^**2**^**)**	E/A ratio	E/e’ ratio	LVFP	Pericard. effusion
1 2 3 4 5 6 7 8 9 10 11 12 13 14 15 16	No No — No No No No No No — No No No No No No	21 24 21 23 21 22 22 23 23 24 22 24 22 21 21 24	3.1 3.3 4.4 3.9 3.5 3.3 3.4 4.8 3.6 2.1 2.0 2.3 2.4 2.6 2.6 2.6	3.2 4.3 3.9 4.9 3.7 4.3 4.7 3.8 3.1 4.2 7.2 5.2 6.1 5.0 6.1 8.0	2.0 2.6 2.8 2.4 2.6 2.6 2.8 2.3 1.6 2.5 4.2 3.0 3.3 3.3 3.3 4.5	11 10 6 4 8 9 6 5 12 14 6 12 7 8 5 3	18 5 2 1 7 4 4 4 5 — 6 7 4 5 2 3	55 68 55 68 — 71 74 — 63 — 79 73 77 60 76 79	— — No No — — — — No — No — — — — No	— — — — 22 29 29 38 — 33 — 40 — 37 — —	— — — — — — 6 6 — 19 — 13 — 13 — —	— — — 28 — — — — 28 37 — NA — — — —	— — — 13 — — — — 17 20 — NA — — — —	2.13 2.70 3.15 3.37 3.2 3.3 — 3.18 2.85 3.05 3.05 2.77 3.1 3.04 3.41 2.0	27 11 12 17 14 13 16 12 22 13 16 20 14 16 20 16	1.2 0.8 0.7 1.0 0.9 0.7 1.1 — 0.8 0.6 0.8 0.8 0.6 0.7 1.1 0.8	— 100 77 60 100 150 43 62 81 37 110 104 114 69 78 120	24 7.5 17 N 15 19 11 15 10 19 12 11 15 16 10 8	20 29 19 21 24 24 22 21 16 21 32 23 30 23 24 29	— — 10 10 15 11 13 17 9.3 — 10.6 — 12.6 — 15 17.2	50 65 45 60 70 65 60 70 60 60 65 70 60 50 65 65	23 16 23 18 36 21 18 18 28 21 — 20 — 16 19 16.5	0.6 0.7 — 0.7 1.1 1.1 0.7 0.8 3.7 1.4 1.0 0.9 1.3 1.0 0.8 0.8	— N N 5.0 7.9 15.2 5.5 6.4 4.9 14.5 — 12.2 — 11.6 — —	N N N N N N N N — — — — — — —	No — No No No — — — No — — — — No — —

Pt #	Screening strategies			Follow-up				
	DETECT algorithm	ESC/ERS echocardiographic probability of PH	ASIG algorithm	ESC/ERS risk assessment at baseline (worsening or death at 1 year)	Number of PH drugs (at baseline - during follow-up)	Duration of follow-up (y)	Occurrence of mPAP > 25 mmHg during follow-up (delay from 1^st^ RHC—months)	Death
1 2 3 4 5 6 7 8 9 10 11 12 13 14 15 16	— RHC recommended — RHC recommended RHC recommended RHC recommended RHC recommended — RHC recommended — — — — RHC recommended — —	— Low Intermediate Intermediate Intermediate Intermediate Intermediate Intermediate Intermediate Intermediate Intermediate Low Intermediate Intermediate High Low	— Positive Positive Positive Positive Positive Positive Positive Positive Positive Positive Negative — Positive — —	High risk Intermediate risk High risk Intermediate risk Intermediate risk Intermediate risk High risk High risk High risk Low risk Intermediate risk Intermediate risk Intermediate risk Intermediate risk Intermediate risk Intermediate risk	0—0 0—0 0—0 1—1 0—0 0—2 0—0 0—1 0—1 0—0 0—2 0—0 0—1 0 -0 0—0 0—3	0.3 2.7 0.9 3.4 3.0 4.9 5.3 1.8 3.2 4.3 8.4 3.0 5.4 4.4 2.8 8.0	— No — Yes (11) Yes (28) Yes (24) Yes (60) Yes (15) Yes (18) — Yes (40) No Yes (60) Yes (50) No Yes (64)	Yes No Yes No Yes No Yes Yes No Yes No Yes No No No No

6MWD: 6-min walking distance, ACA: anti-centromere antibodies, AFA: anti-fibrillarin antibodies, ASIG: Australian Scleroderma Interest Group, ATA: anti-topoisomerase antibodies, BNP: brain natriuretic peptide, CI: cardiac index, CO: cardiac output, CTE: chronic thrombo-embolism, dc: diffuse cutaneous, diag: diagnosis, diam: diameter, DLCO: diffusing capacity for carbon monoxide, ESC/ERS: European Society of Cardiology/European Respiratory Society, ext: extensive, F: female, FEFRPS: first except for RP symptom, FEV1: forced expired volume in 1 min, fluid ch: fluid challenge, FVC: forced vital capacity, HVPG: hepatic venous pressure gradient, ILD: interstitial lung disease, IVC: inferior vena cava, KCO: carbon monoxide transfer coefficient, LA: left atrium, lc: limited cutaneous, lim: limited, LV: left ventricle, LVEF: left ventricular ejection fraction, LVFP: elevated left ventricular filling pressure, M: male, mPAP: mean pulmonary arterial pressure, mRSS: modified Rodnan skin score, N: normal value, NA: not available; no spec: positive antinuclear antibodies without antibody specificity identified, NYHA: New York Heart Association, PAAT: pulmonary artery acceleration time, PAWP: pulmonary arterial wedge pressure, pericard.: pericardial, PH: pulmonary hypertension, pred: predicted value, Pt: patient, PVOD: pulmonary veno-occlusive disease, PVR: pulmonary vascular resistance, RA: right atrium, RAP: right atrial pressure, RAxD: right axis deviation, RHC: right heart catheterization, RHF: clinical signs of right heart failure, RP: Raynaud phenomenon, RV: right ventricle, SSc: systemic sclerosis, SvO_2_: venous saturation in oxygen, TAPSE: tricuspid annular plane systolic excursion, tel: telangiectasias, TLC: total lung capacity, TRV: tricuspid regurgitation velocity, vol: volume, WU: Wood units, y: years; yo: years old

^*^Of note, patient #1 had triscupid valvular disease, making echographic markers of PH unreliable

Most of them were women (14/16, 88%) with a median age of 68 (54–78) years old, a limited cutaneous SSc (13/15, 87%) diagnosed a median of 6.5 (1–6) years before and associated with anti-centromere antibodies (11/15, 73%).

Most patients had CI > 2 L/min/m2 (15/16, 94%), RAP < 8 mmHg (14/15, 93%) and SvO_2_ > 65% (9/13, 69%). As per definition, PAWP was normal in all cases on resting RHC; but patients #9 and #10 elevated their value above 15 mmHg after fluid challenge, suggesting a possible post-capillary component associated. Two out of 15 patients (13%) had extensive ILD based on chest CT-scan involvement, but all of them had FVC values > 70%. Four out of 9 patients (44%) had signs of PVOD, and none showed evidence of chronic thrombo-embolism on chest CT or VQ-scan (Table 2).

PH screening tests and severity markers were inconsistently informative in these patients: 6/13 (46%) had NYHA class III or IV; 12/13 (92%) had 6MWD < 440 m; 3/13 (23%) had abnormal BNP levels; none had right axis deviation on ECG; 10/14 (71%) had DLCO < 60% and FVC (%)/DLCO(%) > 1.6. Similarly, echocardiographic markers of PH were inconstantly present: 11/15 patients (73%) had TRV > 2.8 m/s; 11/15 (73%) had PAAT < 105 ms; 4/16 (25%) had RA area > 18cm^2^; 3/15 (20%) had RV/LV ratio > 1.0; and 1/16 (6%) had IVC diameter > 21 mm with decreased inspiratory collapse. All patients had a normal RV function (as estimated by TAPSE and RV S’ wave) (Table 2).

Interestingly, 10/16 patients (62%) eventually had an increase of mPAP value above or equal to 25 mmHg on a follow-up RHC performed a median of 34 (20–58) months after their baseline evaluation, thus fulfilling the previous definition of precapillary PH; and 3/16 (19%) died shortly after their initial RHC. The 3 remaining patients (19%) had no PH after a 2-year follow-up (Table 2).

### Survival analysis according to PVR values

Using a lower PVR threshold could increase the proportion of pre-capillary PH-reclassified patients but might not capture additional patients with increased mortality. To test the relevance of this new PVR criterion, we studied the relationship between PVR and survival in SSc patients with mPAP > 20 mmHg.

**Fig. 2:**
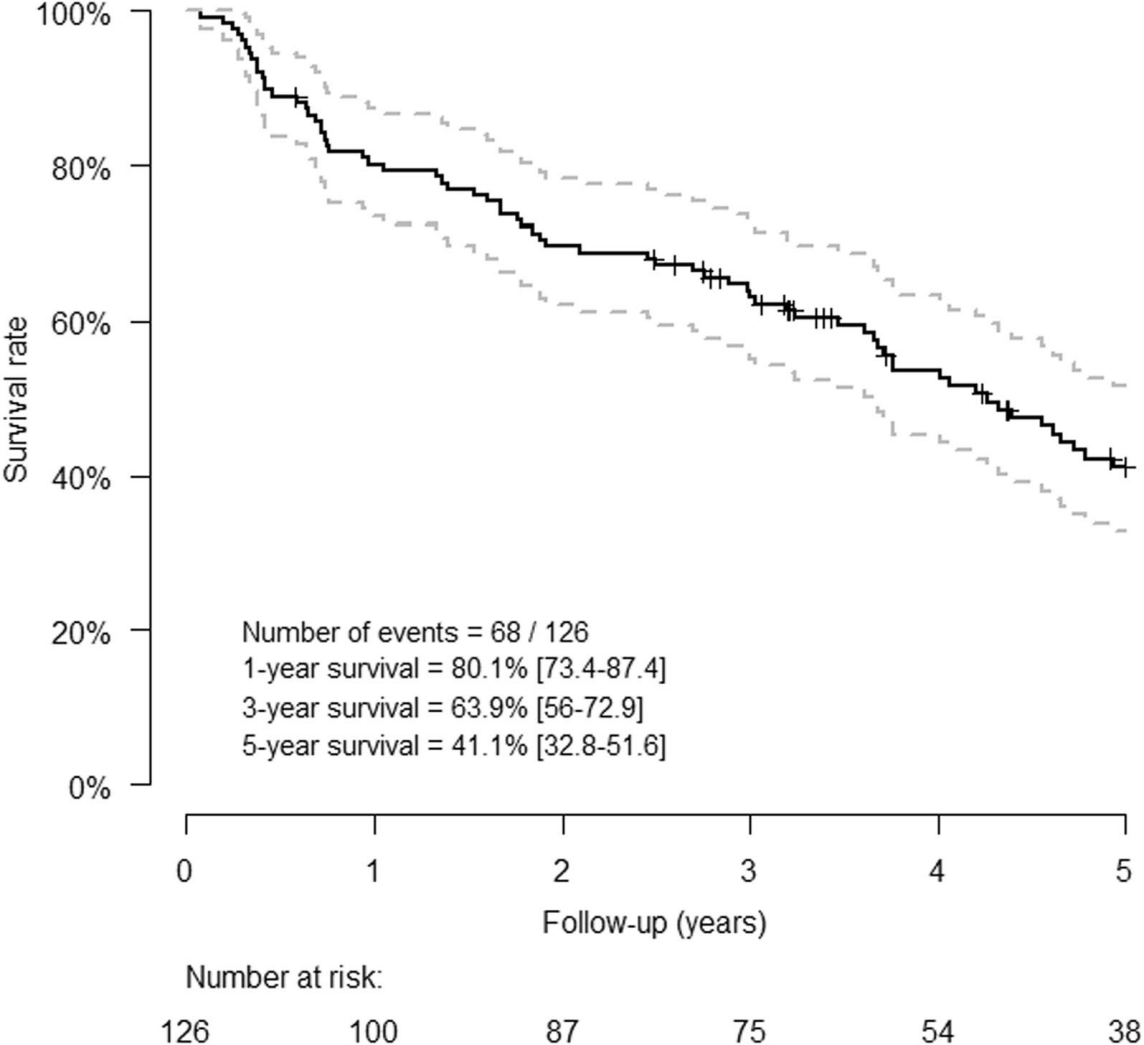
Kaplan-Maier survival curve for the whole population

**Fig. 3:**
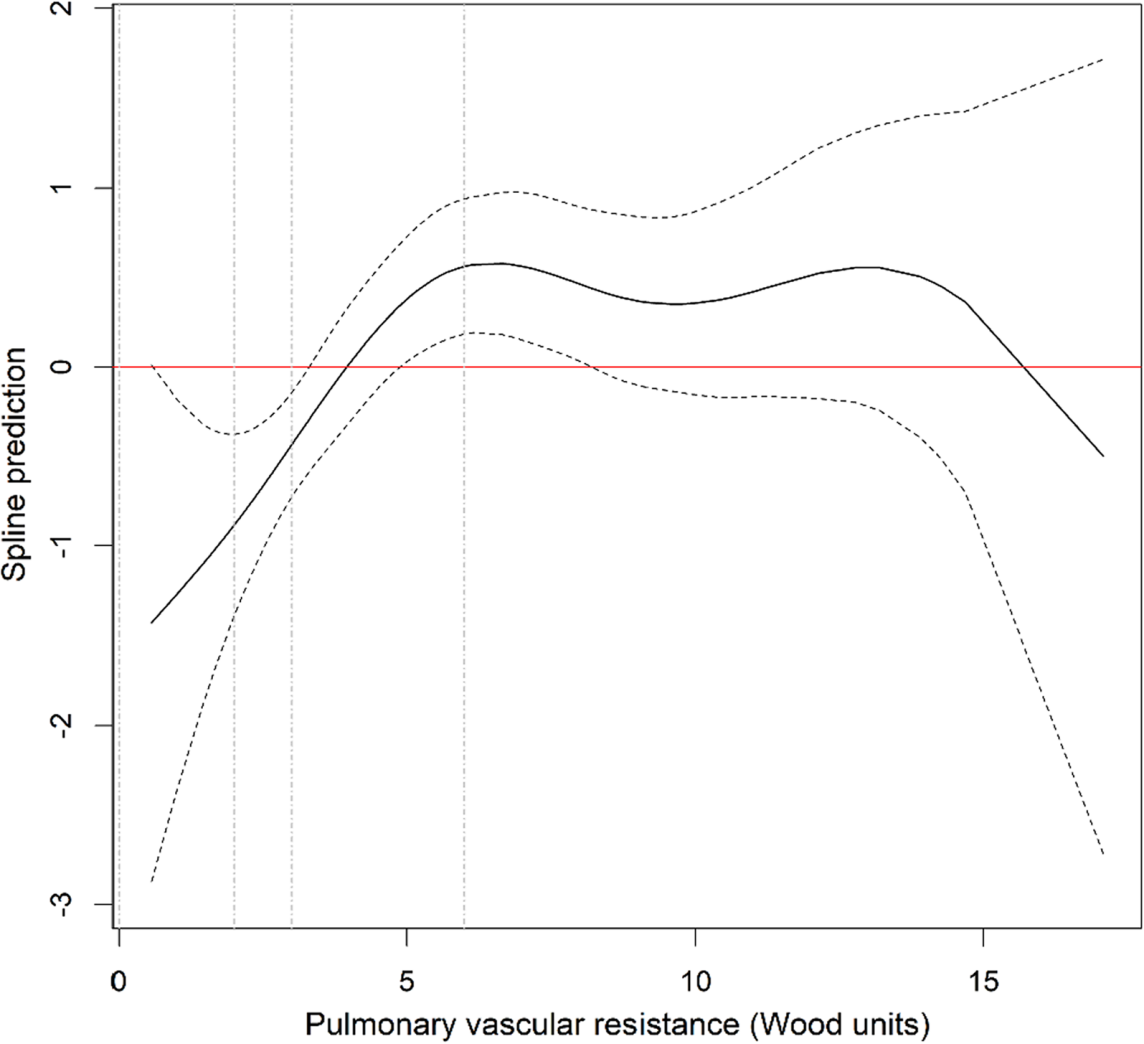
Spline modelling the relationship between survival and PVR values. Spline modelling the relationship between survival and PVR values. The solid line represents the spline prediction (values > 0 indicates an increased risk of mortality, values < 0 a decreased risk of mortality). The dotted curves represent the 95% confidence interval of the spline prediction. The dotted vertical lines represent the 2-WU, 3-WU and 6-WU cut-offs for PVR (from left to right)

**Fig. 4:**
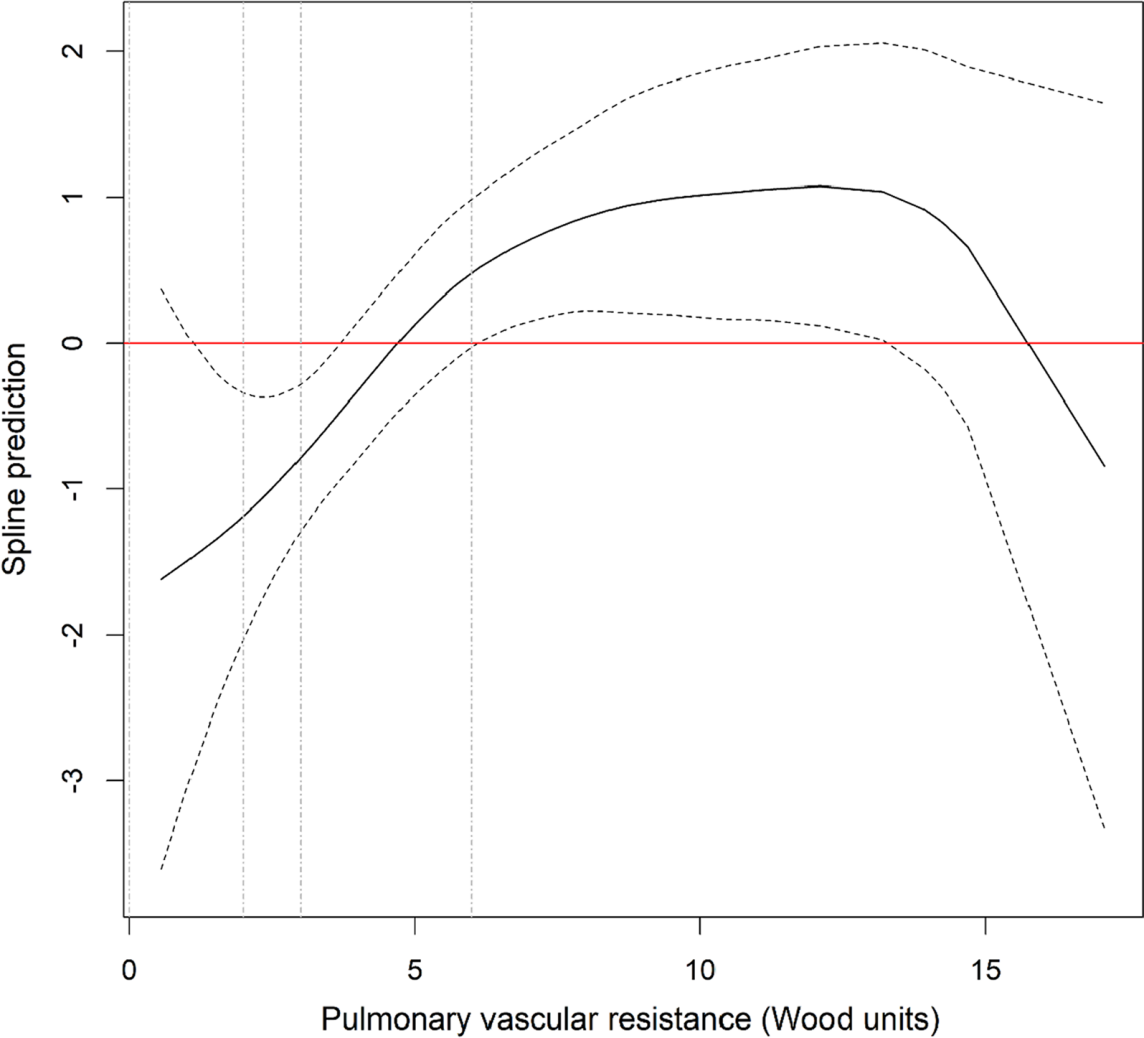
Spline modelling the relationship between survival and PVR values with adjustments for age, gender, SSc subtype, presence of ILD, NYHA functional class and treatment. Spline modelling the relationship between survival and PVR values. The solid line represents the spline prediction (values > 0 indicates an increased risk of mortality, values < 0 a decreased risk of mortality). The dotted curves represent the 95% confidence interval of the spline prediction. The dotted vertical lines represent the 2-WU, 3-WU and 6-WU cut-offs for PVR (from left to right)

The 1-, 3- and 5-year survival rate [95% confidence interval] of the whole population was respectively 80.1% [73.4–87.4%], 63.9% [56–72.9%] and 41.1% [32.8–51.6%] (Fig. 2). In univariate analysis, spline analysis suggests a linear relation between mortality and PVR considering PVR between 0 and 6 WU, and a plateau of mortality for PVR values over 6 WU (Fig. 3). After adjusting on age, sex, SSc subtype, disease duration, presence of ILD, NYHA functional class and treatment, the relation appears similar to the univariate analysis (Fig. 4).

**Fig. 5.**
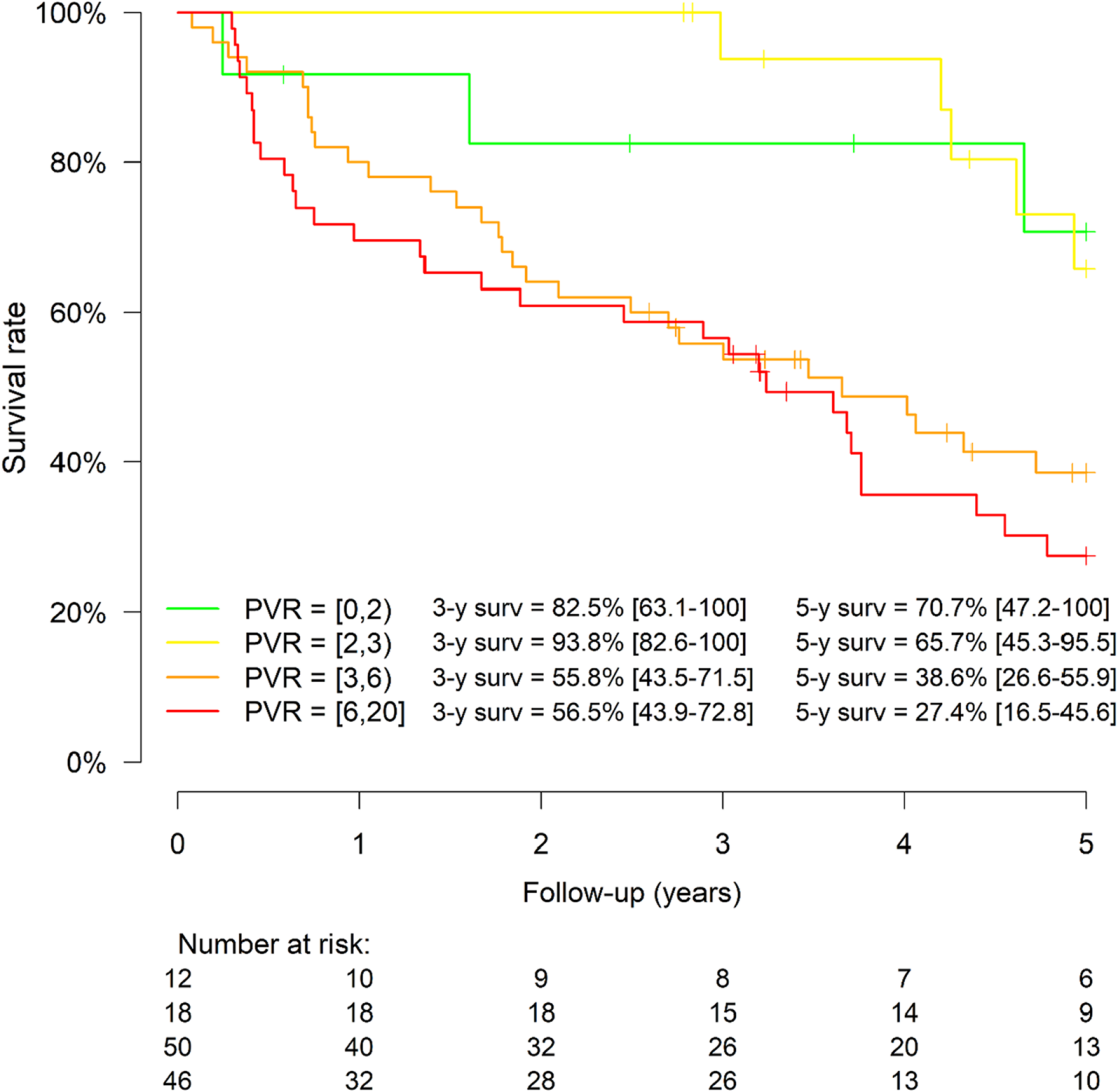
5-year Kaplan-Maier survival curve by PVR groups

Regarding PVR groups, in univariate analysis, the hazard ratio (HR) of death of patients considering their PVR values (reference: PVR < 2WU) were 1.08 [0.2–3.9] for PVR between 2-3WU, 2.9 [0.9–9.4] for PVR between 3-6WU, and 3.7 [1.1–12] for PVR > 6WU (Fig. 5). In multivariate analysis (Additional file 1: Table S1), after adjusting for age, sex, SSc type, disease duration, presence of ILD, NYHA functional class and treatment, HR of death were respectively 0.49 [0.04–5.56], 2.56 [0.5–11.9] and 5.1 [0.97–27] for PVR between 2–3 WU, 3–6 WU and > 6 WU. Presence of ILD (2.36 [1.22–4.6], *p* = 0.02) and triple therapy (0.32 [0.12–0.87], *p* = 0.03) were independently associated with survival in our population. After excluding patients treated by triple therapy to limit survival bias, results were similar (data not shown).

## Discussion

The results of our study can be summarized as follows: 1/ using the new 2022 ESC/ERS definition of PH, 13% of patients were reclassified as having precapillary PH; 2/ at least 62% of them experienced an increase of mPAP values above 25 mmHg during follow-up and eventually met the previous definition of PH; 3/ our spline analysis suggests that mortality increases linearly with PVR values in patients with mPAP > 20 mmHg and PVR < 6 WU.

In our cohort of SSc patients with mPAP > 20 mmHg, 16 (13%) were reclassified as having pre-capillary PH when applying the new criteria. Interestingly, in their work performed in 2 PH centres, Xanthouli et al. identified 28 SSc patients with mPAP 21–24 mmHg and PVR ≥ 2 WU, setting the proportion of reclassified patients at 9.85% when considering their total cohort and 20% when considering only patients with mPAP > 20 mmHg. Other studies found lower proportions of reclassification, ranging from 1.4 to 10.8% [5–7], but since they used the provisional 6^th^ WSPH definition with a PVR cut-off at 3 WU, further comparison with our work is challenging. Coghlan et al*.* analyzed a 3-year follow up of patients with SSc and mPAP < 25 mmHg: 18/71 patients (25%) developed PH during follow-up including 5 with PAH, with an annual incidence of 6.1% which is higher than incidence described in previous cohorts [11]. Considering all PH causes, association between borderline PH and mortality has already been described (with a mPAP threshold of 19 mmHg) [12]. The treatment of patients with mPAP between 21 et 24 mmHg is also still on evaluation. A recent placebo-controlled trial of ambrisentan in SSc PAH patients with mPAP 21–24 failed to show a statistical difference on the primary endpoint but showed interesting results on secondary endpoints (CI and PVR) [13].

There is an ongoing discussion as to whether these new classification criteria capture more patients with decreased survival. When using PVR as a continuous variable, our spline analysis suggested a linear relationship between PVR and survival for PVR values between 0 and 6 WU, implying that patients with PVR > 2 WU may have increased mortality compared to those with lower PVR values. However, when PVR is used as a categorical variable, we did not observe significant differences in survival between PVR 2–3 and PVR 0–2 groups. This discrepancy could be explained by a modest effect size of these low PVR on mortality and by limitations due to our small sample size. In their study performed on 2 large veteran cohorts, Maron et al*.* [14] suggested that the optimal PVR cut-off identifying increased mortality is set at 2.2 WU. Interestingly, when categorizing PVR into 3 groups (with cut-offs at 2.2 and 3.0 WU), the hazard ratio for mortality (reference: PVR 2.2–3.0) was 0.89 (0.80–1.00) (*p* = 0.048) in the PVR < 2.2 group, suggesting an only mildly decreased survival for patients with PVR 2.2–3.0. Conversely, Yamamoto et al*.* [15] did not observe significant differences in the survival of various types of PH patients when comparing PVR < 2 WU and PVR 2–3 WU groups, although their analyses are probably limited by small sample sizes as well. Taken together, these data could suggest an increased mortality at the PVR 2WU cut-off, although it probably remains relatively modest within the 2–3 WU range.

Associations between PVR and mortality have also previously been reported in patients with idiopathic and SSc-associated PH, although with PH defined as mPAP > 25 mmHg [6, 16–18], with a minimum PVR threshold of 4.6 WU. PVR appears to be lower compared to patients with idiopathic PAH, but without correlation with mortality although prognosis of idiopathic PAH is better than in SSc [19, 20] In our cohort, we observed a linear relation between PVR and survival for PVR values < 6 WU, although the increase in mortality seems modest between patients with PVR < 2 WU and PVR between 2–3 WU. When patients with normal or only mildly elevated PVR were excluded, we did not observe a difference in relationship between PVR and CI in patients with SSc-PAH suggesting that increased stiffness of the pulmonary arterial bed is unlikely to be responsible for differences in outcomes. The patients with PVR between 2 and 3 also appear to be at risk of progression to PH [21], which seems to be also observed in our cohort.

Our study has several limitations. RHC was only performed in patients with a suspicion of PH, leading to selection bias with patients with milder hemodynamic alterations which may have been underdiagnosed; and RHC indications have changed during the inclusion period. Our study is also limited by its retrospective single-center design and its small sample sizes, although it includes a rather large cohort of SSc patients with hemodynamic evaluation. Our population may be heterogeneous as it mixed patients with different mechanisms of PH (groups 1 and/or 2 and/or 3) and thus possibly different prognosis; however, this remains relevant as all patients with mPAP ≥ 20 mmHg and PVR ≥ 2 WU are considered to have at least pre-capillary PH (with or without an associated post-capillary PH, depending on their PAWP value) and some degree of pulmonary microangiopathy. Moreover, in SSc patients with ILD and PH, it is often difficult to determine whether PH is exclusively due to the chronic lung disease (group 3 only) or also caused by an underlying microangiopathy (group 1 + 3). Finally, multivariate analysis must be taken with caution as the adjustment is limited by the small effectives and by missing data for several predetermined variables.

## Conclusion

A significant proportion of SSc patients is reclassified as having pre-capillary PH with the new 2022 ESC/ERS hemodynamic definition. Lowering the PVR threshold from 3 to 2 WU captures patients at risk of raising their mPAP > 25 mmHg, with a possibly less severe disease. Prospective studies on larger cohorts are warranted to draw firm conclusions on the impact of this new definition.

## Supplementary Information

**Additional file 1:** Multivariate analysis after adjusting for age, sex, SSc type, disease duration, presence of ILD, NYHA functional class and treatment.

## Data Availability

The datasets used and/or analyzed during the current study are available from the corresponding author upon reasonable request from any qualified researcher.
